# An Uncommon Case of Recurrent Spontaneous Coronary Artery Dissection Involving Multiple Coronary Arteries

**DOI:** 10.7759/cureus.108885

**Published:** 2026-05-15

**Authors:** Muhammad Asif, Talha Shamshad, Sukhila Reddy, Osama Alsara, Suneel Kumar

**Affiliations:** 1 Cardiology, Carle Foundation Hospital, Urbana, USA; 2 Internal Medicine, Carle Foundation Hospital, Urbana, USA

**Keywords:** acs in females, cardiac chest pain in young, management of scad, recurrent spontaneous coronary artery dissection, stemi which may not need pci

## Abstract

Spontaneous coronary artery dissection (SCAD) is an uncommon cause of cardiac ischemia that especially affects young women. Its true prevalence is not well established due to limitations in diagnosis. Although the symptoms of SCAD largely mimic those of acute coronary syndrome (ACS), the management is remarkably different. The etiology of SCAD is considered to be multifactorial, and underlying arteriopathic disease, such as fibromuscular dysplasia, is a well-known risk factor. We treated a case of a young woman who had recurrent SCAD involving three different coronary arteries, including the left anterior descending, right coronary artery, and obtuse marginal branch, on two different occasions without any identifiable underlying vascular disease or acute precipitant. She had STEMI (ST-segment elevation myocardial infarction) on her first presentation and NSTEMI (non-ST segment elevation myocardial infarction) on her second presentation nearly two years later. She underwent angiography on both occasions; however, she was treated medically without percutaneous coronary intervention (PCI) and had good outcomes with the resolution of symptoms.

## Introduction

Spontaneous coronary artery dissection (SCAD) is a known etiology of acute coronary syndrome (ACS). Most of the patients diagnosed with SCAD are women under the age of 50 years, without having conventional risk factors of atherosclerotic cardiovascular disease (ASCVD). Although SCAD results in symptoms similar to type 1 myocardial infarction (MI) due to the difference in the underlying mechanism of injury, the acute and long-term management of ischemia caused by SCAD is remarkably different [1]. The evidence for secondary prevention is still evolving. Therefore, reporting and dissemination of more patient data and outcomes from unique presentations can inform future research and the development of guidelines for the diagnosis and management of SCAD. We present a case of recurrent SCAD involving three different vessels, successfully managed with a conservative approach.

## Case presentation

**Figure 1:**
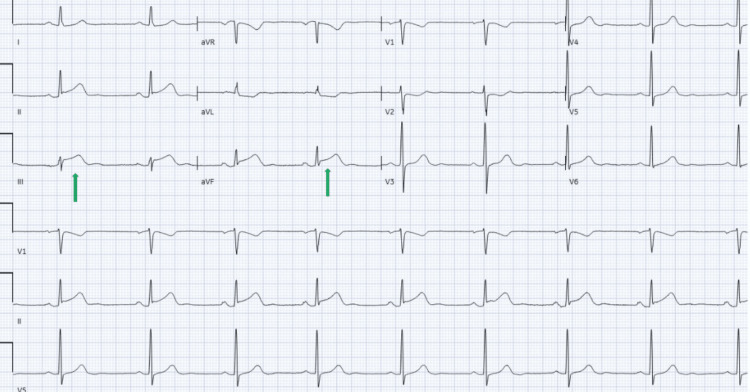
EKG on first presentation: Green arrows indicate ST segment elevations in leads III and augmented vector foot (aVF).

A 41-year-old woman who presented with a chief complaint of precordial chest pain radiating to the left arm. The pain woke her up from sleep, was intermittent in nature, moderate in intensity, and was associated with diaphoresis and an episode of emesis. Her past medical history (PMH) included hypertension (HTN), anxiety, and obesity. No family history of coronary artery disease (CAD), and she denied alcohol or drug abuse. She also had a previous inferior ST-segment elevation myocardial infarction (STEMI) (Figure 1) two years before the index presentation due to SCAD of the obtuse marginal (OM2) branch of the left circumflex coronary artery diagnosed by left heart catheterization (LHC). No percutaneous coronary intervention (PCI) was indicated during her prior SCAD, as she was hemodynamically stable and no major coronary territory was involved, and she had thrombolysis in myocardial infarction grade 2 (TIMI 2) flow in the affected OM2 branch.

**Figure 2:**
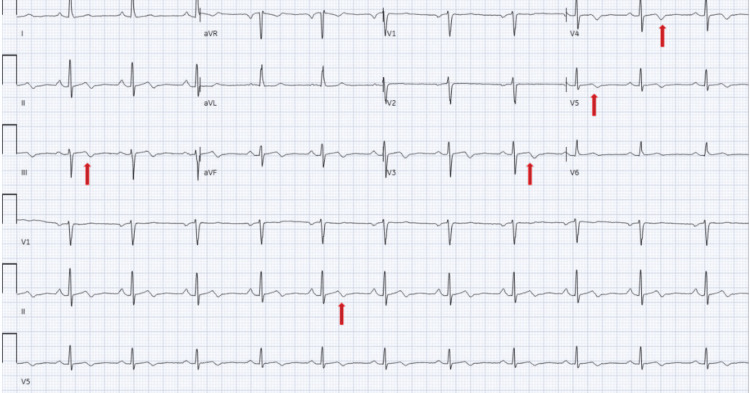
EKG: Red arrows indicating T wave abnormalities in the inferior and chest leads.

Her home medications included aspirin 81 mg daily, atorvastatin 40 mg daily, and metoprolol succinate 25 mg daily, with suboptimal compliance. In the emergency room (ER), her vital signs were: blood pressure (BP) 148/87 mmHg, heart rate (HR) 77 bpm, respiratory rate (RR) 25/min, peripheral oxygen saturation (SpO_2_) 96%. The physical exam was unremarkable. Labs were notable for abnormal high-sensitivity troponin levels of 184 and 157 ng/L (reference value 0-4 ng/L). EKG on this presentation revealed new T-wave abnormalities in the inferior, anteroseptal, and lateral chest leads (Figure 2).

**Figure 3:**
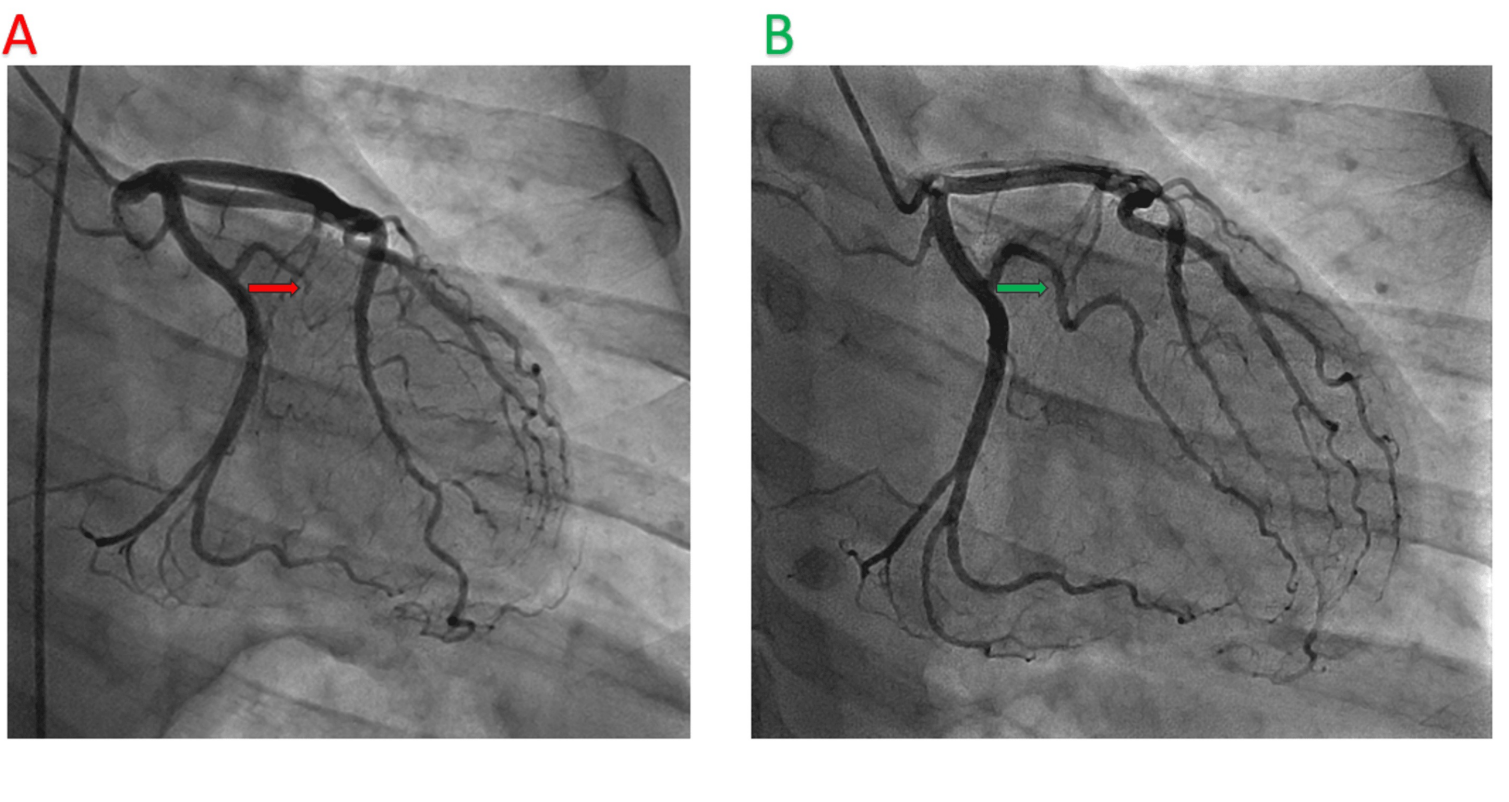
Angiographic views of the coronary arteries. A: Red arrow indicating spontaneous coronary artery dissection (SCAD) of the obtuse marginal 2 (OM2) branch on previous angiography. B: Green arrow indicating resolution of the OM2 SCAD on the second angiography with thrombolysis in myocardial infarction grade 3 (TIMI) 3 flow.

**Figure 4:**
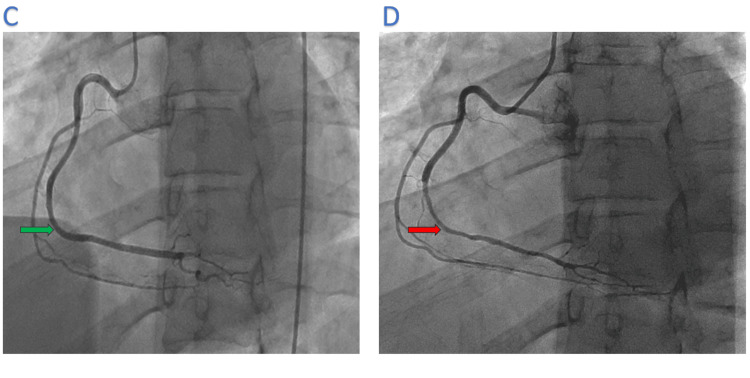
Angiographic views of the coronary arteries. C: Green arrow indicating normal caliber of the RCA on previous angiography. D: Red arrow indicating new SCAD of the RCA on the second angiography.

**Figure 5:**
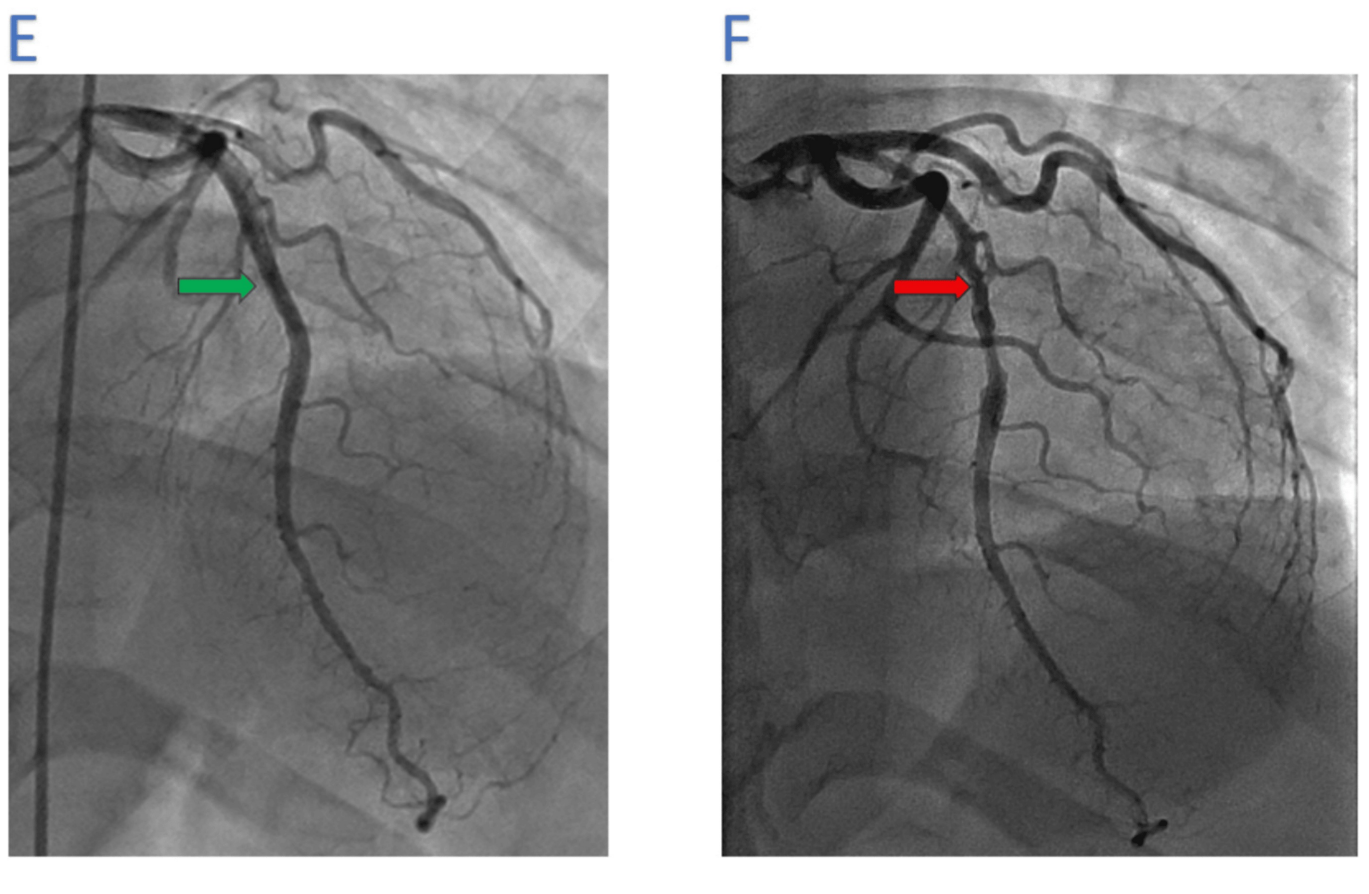
Angiographic views of the coronary arteries. E: Green arrow indicating normal caliber of the LAD on previous angiography. F: Red arrow indicating new SCAD of the LAD on the second angiography.

She was diagnosed with non-ST-segment elevation myocardial infarction (NSTEMI) and started on aspirin, heparin infusion, and nitroglycerin; she was admitted to the hospital. Her chest pain continued to recur; therefore, she underwent urgent left heart catheterization, which revealed that the distal right coronary artery (RCA) and right posterior descending artery (RPDA) had a diffuse luminal taper, causing 50% stenosis, consistent with type 2 spontaneous coronary artery dissection. Left anterior descending artery (LAD) also had a tapered, diffuse 50%-60% stenosis in the proximal to mid portion, consistent with type 2 SCAD. The OM2 that previously had SCAD had completely healed with TIMI 3 flow (Figure 3B). Although these abnormalities in the RCA and LAD were new compared with the previous angiogram (Figures 4C, 5E), the patient was hemodynamically stable with adequate distal flow; therefore, no PCI was performed. Anticoagulation was discontinued; however, aspirin, clopidogrel, and metoprolol were continued.

Although the possibility of recurrent SCAD was in the differential diagnosis, the patient was sent for left heart catheterization (LHC) due to ongoing chest pain of a different nature and location than her previous myocardial infarction (MI), along with new abnormalities in the EKG and elevated high-sensitivity troponin. The patient was monitored for 72 hours inpatient due to a high risk of complications in SCAD-related MI. She was started on amlodipine for optimization of blood pressure and secondary prevention. Her chest pain resolved, vital signs remained stable during the rest of her hospital course, and she was eventually discharged home.

Since SCAD is known to have an association with arteriopathies, including fibromuscular dysplasia (FSD), as an outpatient, she underwent CT angiography (CTA) head and neck CTA, CTA of the chest, abdomen, and pelvis, which were negative for any occlusive arterial disease, aneurysm, or dissection. The patient was also referred to for rehab and genetic counseling, where she had good activity tolerance and decided not to pursue a future pregnancy. She followed up with the cardiology office and, at six months, remained asymptomatic with good functional capacity.

## Discussion

SCAD is defined as a dissection of an epicardial coronary artery that is not associated with atherosclerosis and is not caused by trauma or iatrogenic etiology. The exact incidence is not known; various studies have shown that SCAD may be a cause of up to 1% to 4% of ACS cases overall, and it occurs predominantly in women. It is the cause of ACS in up to 35% of MIs in women ≤50 years of age, and is the most common cause of pregnancy-associated MI reported up to 43% by one study [1,2].

The hallmark of SCAD is the spontaneous formation of an intramural hematoma within the wall of a coronary artery. There are two theories about the mechanism of SCAD development [3]. The first theory postulates that the primary event is the intimal tear, which allows blood from the true lumen to enter and create a false lumen within the vessel wall. The second theory suggests that the primary pathologic event is a spontaneous hemorrhage arising from the vasa vasorum within the vessel wall. The latter theory is supported by the fact that intimal tear is not identified in many patients diagnosed with SCAD, even with intracoronary imaging [4].

Because of the unique epidemiology and the low prevalence of traditional cardiovascular risk factors, the etiology of SCAD is hypothesized to be multifactorial, including genetics, acquired or inherited arteriopathies, hormonal factors, or systemic inflammatory diseases, often superimposed by environmental precipitating stressors [1].

Fibromuscular dysplasia (FMD) of the extracoronary arteries is the most commonly reported arteriopathy associated with SCAD. FMD is a nonatherosclerotic, noninflammatory vascular disease that can involve nearly any arterial bed and can manifest as arterial stenosis, aneurysm, tortuosity, or dissection [5]. The most commonly reported precipitants of SCAD are extreme physical (24%) or emotional (40%) stress reported to occur before the event. The most common symptom is chest pain (95.9%) and its radiation to the arm (51.5%) [6].

If SCAD is clinically suspected, coronary angiography should be performed, especially in the setting of STEMI. Coronary angiography remains the first-line diagnostic imaging method despite its limitations because it is widely available and recommended for early invasive management of ACS, along with as-needed intracoronary imaging methods, including intravascular ultrasonography and optical coherence tomography. Angiographically, SCAD is classified into types 1-4. Type 2 SCAD is the most common and appears as a long, smooth stenosis [7].

The majority of patients with SCAD heal spontaneously within a few weeks, with rates of 70%-90% [1]. In one observational study, 131 SCAD lesions were followed with angiography after 35 days, and healing was observed in all the cases [8]. This, along with risks of additional diagnostic procedures, favors the approach that, in the absence of any symptoms, repeat angiography or coronary computed tomography angiography (CCTA) may not be needed to confirm healing despite conservative management. On the other hand, conservative therapy may not be appropriate in high-risk patients with ongoing ischemia, left main artery dissection, or hemodynamic instability. In such cases, urgent PCI or coronary artery bypass grafting (CABG) should be considered on an individual basis and based on the patient's coronary anatomy. Irrespective of the treatment strategy, all patients having MI due to SCAD should be hospitalized for 48-72 hours to monitor for complications, recurrence, or progression of the disease [9].

The evidence for the best medical treatment for SCAD patients is still evolving and largely based on expert opinion and consensus statements. A few aspects that contrast its management from the typical atherosclerotic ACS are cautious approach to the use of dual antiplatelet (DAPT) since no RCT has compared outcomes or bleeding risks related to the use of dual-antiplatelet therapy and aspirin alone in SCAD and considering discontinuation of systemic heparin once the diagnosis of SCAD has been made in the absence of another indication for these due to risk from bleeding complications [1,10].

The use of DAPT is often extrapolated from the ACS recommendations, and different experts have reported using it for 12 months, followed by lifelong aspirin or DAPT for three to 12 months, followed by aspirin alone [11]. However, in observational studies, there was no significant difference in bleeding risk or outcomes between patients treated conservatively or with PCI after SCAD, regardless of DAPT duration [12]. Many experts support beta-blocker use in SCAD patients, supported by a study showing a hazard ratio of 0.36 for recurrent SCAD at 3.1-year follow-up [6].

Patients who wish to have a pregnancy after SCAD should be referred to tertiary medical centers for preconception assessment and counseling and should be counseled on the potential increased risk of dissection and complications during the pregnancy. Given the high co-prevalence of FMD, aneurysm, and other extra-coronary vascular abnormalities among patients with SCAD, a thorough family history, clinical exam for vascular and connective tissue abnormalities, and vascular imaging from the brain to the pelvis should be considered in all patients, as is current practice for FMD [5]. The role of genetic testing counseling is unclear.

Our case had many of these typical risk factors, including female gender, age less than 50, a history of anxiety, and no known atherosclerotic disease before. She was unique in the sense that no specific stressor could be identified, and had three different vessels involved and different symptom characteristics during the two episodes of SCAD presentations. Future prospective larger-scale randomized and epidemiological studies can help increase our understanding of the demographics, management, and anatomic factors associated with recurrence and aid in more accurate prediction and prevention of recurrent SCAD.

## Conclusions

This young woman, who had her second acute coronary event related to SCAD involving three vessels in two different presentations, was successfully treated without PCI. We focused on secondary prevention through close inpatient monitoring, BP optimization, screening for fibromuscular dysplasia, and genetic counseling to identify and mitigate any associated risk factors.
